# Proteomics analysis reveals novel host molecular mechanisms associated with thermotherapy of ‘*Ca.* Liberibacter asiaticus’-infected citrus plants

**DOI:** 10.1186/s12870-016-0942-x

**Published:** 2016-11-14

**Authors:** Chika C. Nwugo, Melissa S. Doud, Yong-ping Duan, Hong Lin

**Affiliations:** 1 USDA, Agricultural Research Service, San Joaquin Valley Agricultural Sciences Center, 9611 South Riverbend Avenue, Parlier, 93648 CA USA; 2 USDA, Agricultural Research Service, U.S. Horticultural Research Laboratory, Fort Pierce, 34945 FL USA

**Keywords:** Citrus, Proteomics, Huanglongbing, Host response, Protein extraction, Chaperones, Defense-related proteins, Heat treatment, Photosynthesis-related proteins

## Abstract

**Background:**

Citrus Huanglongbing (HLB), which is linked to the bacterial pathogen ‘*Ca.* Liberibacter asiaticus’ (Las), is the most devastating disease of citrus plants, and longer-term control measures via breeding or genetic engineering have been unwieldy because all cultivated citrus species are susceptible to the disease. However, the degree of susceptibility varies among citrus species, which has prompted efforts to identify potential Las resistance/tolerance-related genes in citrus plants for application in breeding or genetic engineering programs. Plant exposure to one form of stress has been shown to serendipitously induce innate resistance to other forms of stress and a recent study showed that continuous heat treatment (40 to 42 °C) reduced Las titer and HLB-associated symptoms in citrus seedlings. The goal of the present study was to apply comparative proteomics analysis via 2-DE and mass spectrometry to elucidate the molecular processes associated with heat-induced mitigation of HLB in citrus plants. Healthy or Las-infected citrus grapefruit plants were exposed to room temperature or to continuous heat treatment of 40 °C for 6 days.

**Results:**

An exhaustive total protein extraction process facilitated the identification of 107 differentially-expressed proteins in response to Las and/or heat treatment, which included a strong up-regulation of chaperones including small (23.6, 18.5 and 17.9 kDa) heat shock proteins, a HSP70-like protein and a ribulose-1,5-bisphosphate carboxylase oxygenase (RuBisCO)-binding 60 kDa chaperonin, particularly in response to heat treatment. Other proteins that were generally down-regulated due to Las infection but up-regulated in response to heat treatment include RuBisCO activase, chlorophyll a/b binding protein, glucosidase II beta subunit-like protein, a putative lipoxygenase protein, a ferritin-like protein, and a glutathione S-transferase.

**Conclusions:**

The differentially-expressed proteins identified in this study highlights a premier characterization of the molecular mechanisms potentially involved in the reversal of Las-induced pathogenicity processes in citrus plants and are hence proposed targets for application towards the development of cisgenic Las-resistant/tolerant citrus plants.

**Electronic supplementary material:**

The online version of this article (doi:10.1186/s12870-016-0942-x) contains supplementary material, which is available to authorized users.

## Background

Citrus Huanglongbing (HLB) is considered the most devastating disease threatening citrus production worldwide [1, 2]. The disease, which was first discovered in Asian countries in the 1870s, is now prevalent in many citrus growing regions in the world including the U.S.A., Brazil, Iran and Saudi Arabia [2]. In the U.S., HLB has cost the state of Florida over $3.5 billion in lost revenue since its initial incidence in 2005 [3]. The disease is now looming large for California and Texas, two major citrus production states in U.S. [4, 5].

Although Koch’s postulate has yet to be fulfilled, HLB is etiologically-linked to three species of insect-transmissible fastidious, phloem-restricted α-proteobacteria: ‘*Candidatus* Liberibacter asiaticus’ (Las), ‘*Ca*. L. africanus’ (Laf), and ‘*Ca*. L. americanus’ (Lam) [2]. Among these three Liberibacter species, Las has the largest geographical distribution and is the species present in the U.S. [1, 2]. Las is disseminated naturally by the Asian citrus psyllid *Diaphorina citri* Kuwayama (Hemiptera: Psyllidae) [6]. Feedings by the insect result in simultaneous transmission of the bacteria to the phloem [7]. Infected trees show gradual but irreversible decline within a few years post-infection and growers currently lack practical options to combat the pathogen besides removal of infected trees to prevent spread to other trees [1]. A common preventive approach is the use of insecticides, which have to be applied multiple times a year to suppress psyllid populations [2]. However, the steep financial burden concomitant with current HLB control measures, especially for small scale growers, coupled with the increased incidence and severity of HLB around the world underscores the need for more effective control measures.

Thermal therapy treatments have been used for decades against plant infections and there are reports as early as 1936 showing the use of dry heat and hot-water treatments to eliminate peach yellows and other chlorotic diseases caused by viral infections [8]. Heat treatment has been used to prevent or cure multiple plant diseases including ratoon stunting disease of sugarcane caused by *Leifsonia xyli* [9, 10] and citrus quick-decline disease caused by *Citrus tristeza* [11–13]. Recently, Hoffman et al. [14] demonstrated that continuous thermal exposure of 40 to 42 °C for time periods ranging from 2 to 7 days markedly reduced Las titer in HLB-affected citrus seedlings. Additionally, Yang et al. [15] showed that effective application of antimicrobial compounds and thermotherapy (chemo-thermotherapy) mitigated HLB in citrus plants. However, unlike chemotherapy, the molecular mechanisms associated with thermotherapy-mediated HLB suppression are unresolved.

Virtually all bacteria, including plant-associated bacteria, are suggested to have prophages incorporated in their genomes [16], which is consistent with the discovery of two prophages in the Las genome that can become lytic during periods of infection [17]. Heat has been shown to induce the lytic cycles of many prophages, including *Escherichia coli* and *Xylella fastidiosa* prophages, resulting in the rapid destruction of bacterial cells [18, 19]. Thus, Hoffman et al. [14] suggested that heat-mediated induction of the lytic cycles of Las prophages could play a role in heat-induced elimination of Las in HLB-affected lemon plants. However, Wang et al. [20] showed that in tobacco (*Nicotiana tabacum*) and *Arabidopsis* plants, the hypersensitive response- and *R*-gene-mediated defense responses to *Pseudomonas syringae* and viral elicitors are compromised at high temperatures, allowing increased pathogen growth. This suggests that heat-induced bacterial pathogen-resistance in plants, as observed in Citrus-Las interactions, could be more complex than earlier thought and other heat-inducible processes, besides prophage cycles, might be involved.

An increasing volume of evidence from field, laboratory and molecular studies suggest that rather than being additive, the presence of an abiotic stress, such as heat, can have the effect of reducing or enhancing susceptibility to a biotic pest or pathogen, and vice versa [21]. For example, in maize, breeding programs for drought tolerance have serendipitously led to plants which are resistant to the parasitic weed *Striga hermonthica* [22, 23]. In wheat (*Triticum aestivum*), higher mean temperatures observed over a 6-year experimental period correlated with increased susceptibility to the fungus *Cochliobolus sativus* [24].

Long-term control of HLB inevitably depends on the development of resistant or tolerant citrus varieties via breeding and genetic engineering programs. Unfortunately, this process is handicapped by the fact that all known citrus species are susceptible to HLB and no readily available genes or sources of resistance have been identified. However, the differences in HLB susceptibility across citrus species, particularly the high tolerance of lemon plants to Las [25], suggest that there are potential innate HLB resistance- and/or tolerance-associated mechanisms in citrus plants. Additionally, the full recovery from pathogen-associated symptoms observed in HLB-affected citrus plants after heat treatment [14], which as earlier mentioned, is not typical for all bacterial-infected plants [20], suggests that heat exposure could induce novel host defense-related mechanisms that suppress pathogen growth [21].

Hence, although shown to be effective in the control of HLB in nursery and greenhouse settings, thermal therapy is currently not practical for trees in the field [14]. Thus, it is hypothesized that a feasible way of applying thermal therapy to field plants would involve the first step of identifying the potential molecular mechanisms/conditions associated with thermal-induced HLB mitigation, which would lead to the downstream development of plants that can mimic those processes in the field.

Additionally, due to the lack of known Las resistance genes in cultivated *Citrus* spp., majority of the crop development research efforts have been geared towards generating transgenic Las-resistant citrus plants. For example, by incorporating multiple copies of naturally occurring spinach defensin genes into citrus plants, Mirkov and Gonzalez-Ramos reportedly developed transgenic citrus plants that are fully or highly resistant to HLB [26]. Although transgenesis and cisgenesis both involve similar highly controversial genetic modification techniques, cisgenesis has better promise towards consumer acceptability because it involves the introduction of genes from the plant or from a close relative, and these genes could also be transferred by traditional breeding techniques [27]. Thus, the goal of this study is to employ a proteomics approach to elucidate the global molecular mechanisms involved in the response of Las-infected citrus plants upon heat exposure. The present study constitutes the first report involving the application of a proteomics approach to elucidate the global molecular mechanisms associated with heat-induced Las-resistance in citrus plants. It is anticipated that the information generated from the present study would assist in the development of cisgenic Las resistant or tolerant citrus plants.

## Results and discussion

### Heat-induced reduction of Las titer

**Fig. 1:**
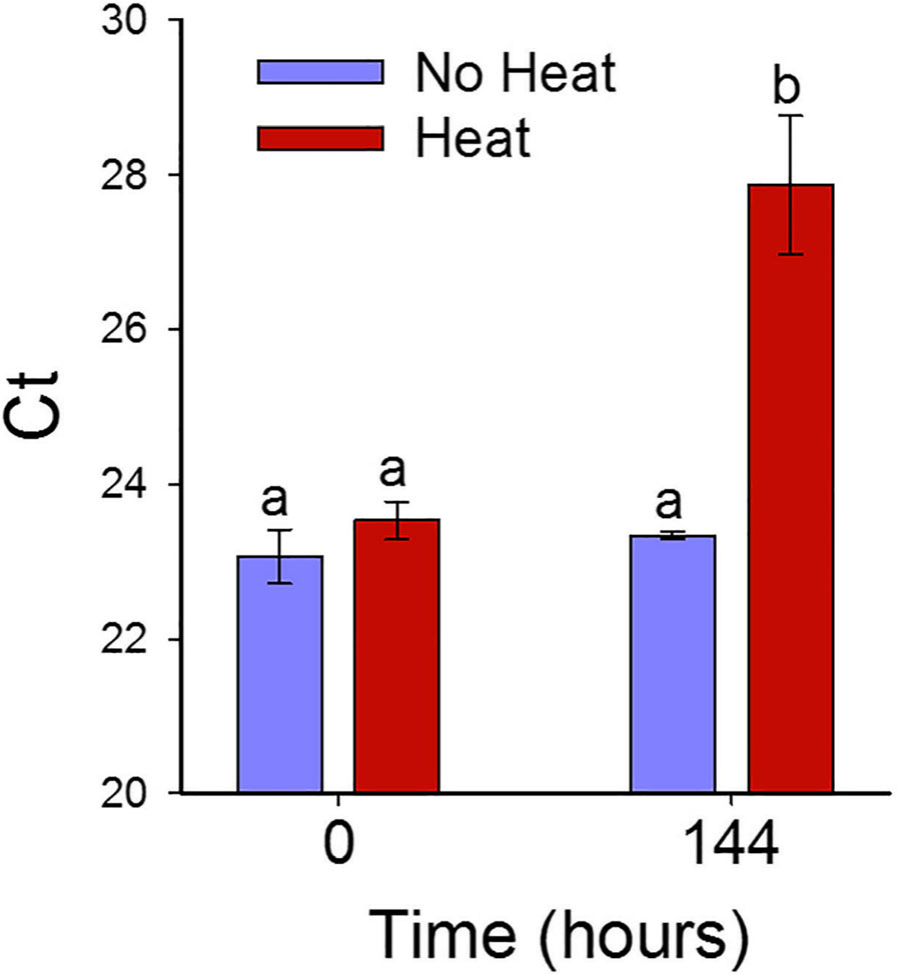
Bar graph showing the relative Las titer in leaves of Las-infected citrus plants grown at room temperature and after exposure to heat treatment. Leaves were harvested at two time points namely; 0 h (before commencement of heat treatment) and 144 h (6 days after commencement of heat treatment). Las titer was measured via qPCR and a lower Ct value denotes higher bacterial titer in leaves. Bars with the same lower case letter were not significantly different from each other

A preliminary screen for Las presence in plant leaves using conventional PCR produced Las-positive bands in ^+^Las/^−^Heat plants and ^+^Las/^+^Heat plants but not in ^−^Las/^−^Heat or ^−^Las/^+^Heat plants, which confirmed the presence of Las only in infected plants. Further analysis via qRT-PCR to compare the effect of heat treatment on Las titer in infected plants tissues showed a significant increase in the mean Ct values of Las infected plant tissues in the presence of heat treatment from 23.53 at time 0 h to 27.86 at time 144 h (Fig. 1). These results are consistent with those from Hoffman et al. [14] and Yang et al. [15], which showed thermotherapy-induced reduction in Las titer of HLB-affected citrus plants.

### Both Las infection and heat treatment confer significant effects on citrus leaf proteomics

The exhaustive total protein extraction method (see Methods section) used in this study produced an average protein yield of over 20 mg g^−1^ from citrus grapefruit leaves irrespective of Las or heat treatment (Table 1), which is higher than the mean protein yield of approximately 13 mg g^−1^ from citrus grapefruit leaves in our prior study [28]. This result validates the efficacy of the total protein extraction method used in the current study. Additionally, using PDQuest gel-image analysis software, the mean number of detected spots was over 1250 in the present study (Table 1), compared to less than 800 in our prior study [28]. Thus, the higher protein yields and improved protein coverage observed in the present study compared to our prior study is encouraging and suggests a more exhaustive comparative proteomics analysis. However, it is important to note that physiological factors including plant age and developmental stage may play a role in the observed differences in protein yield/coverage between our present and earlier studies on citrus leaves and further experimentation is anticipated to fully validate our enhanced total protein extraction method.

**Table 1:** Comparative analysis of the effect of heat treatment on the total leaf proteome of healthy or Las-infected lemon plants. Data represents Means ± SD

Parameters	Treatments			
	^−^Las/^−^Heat	^+^Las/^−^Heat	^−^Las/^+^Heat	^+^Las/^+^Heat
Protein yield^a^ (mg g^−1^)	29.6 ± 5.3	28.8 ± 4.9	21.7 ± 4.1	25.5 ± 4.3
Number of detected spots	1258 ± 17	1305 ± 6	1334 ± 14	1303 ± 19
Number of matched spots in replicate gels	749 ± 15	755 ± 15	883 ± 20	834 ± 15
Number of matched spots to all gels	359	359	359	359

^a^Protein extraction was repeated three times per sample with three replicate plants per treatment

**Fig. 2:**
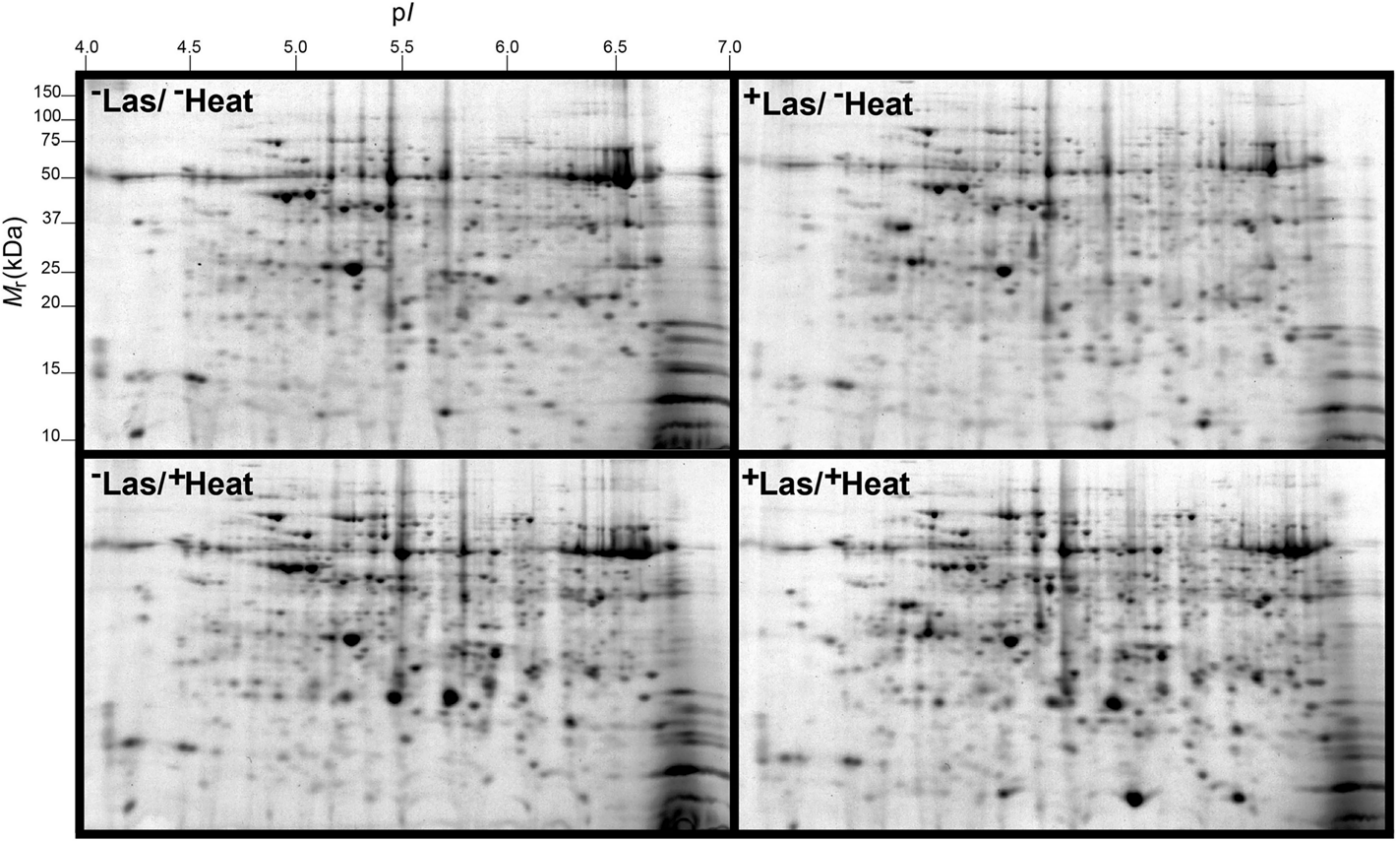
Representative two-dimensional electrophoresis (2-DE) gel maps of total leaf proteome of grapefruit leaves extracted from ^−^Las/^−^Heat, ^+^Las/^−^Heat, ^−^Las/^+^Heat or ^+^Las/^+^Heat plants. Three-year-old similarly-sized healthy or Las-infected plants were either unexposed or exposed to thermal treatment of 40 °C for 6 days in a growth chamber. A total of 300 μg of protein was loaded on a pH 4–7 IpG strip and protein spots were visualized by staining with Coomassie Brilliant Blue (CBB). *M*_r_, relative molecular weight; p*I*, isoelectric point

**Fig. 3:**
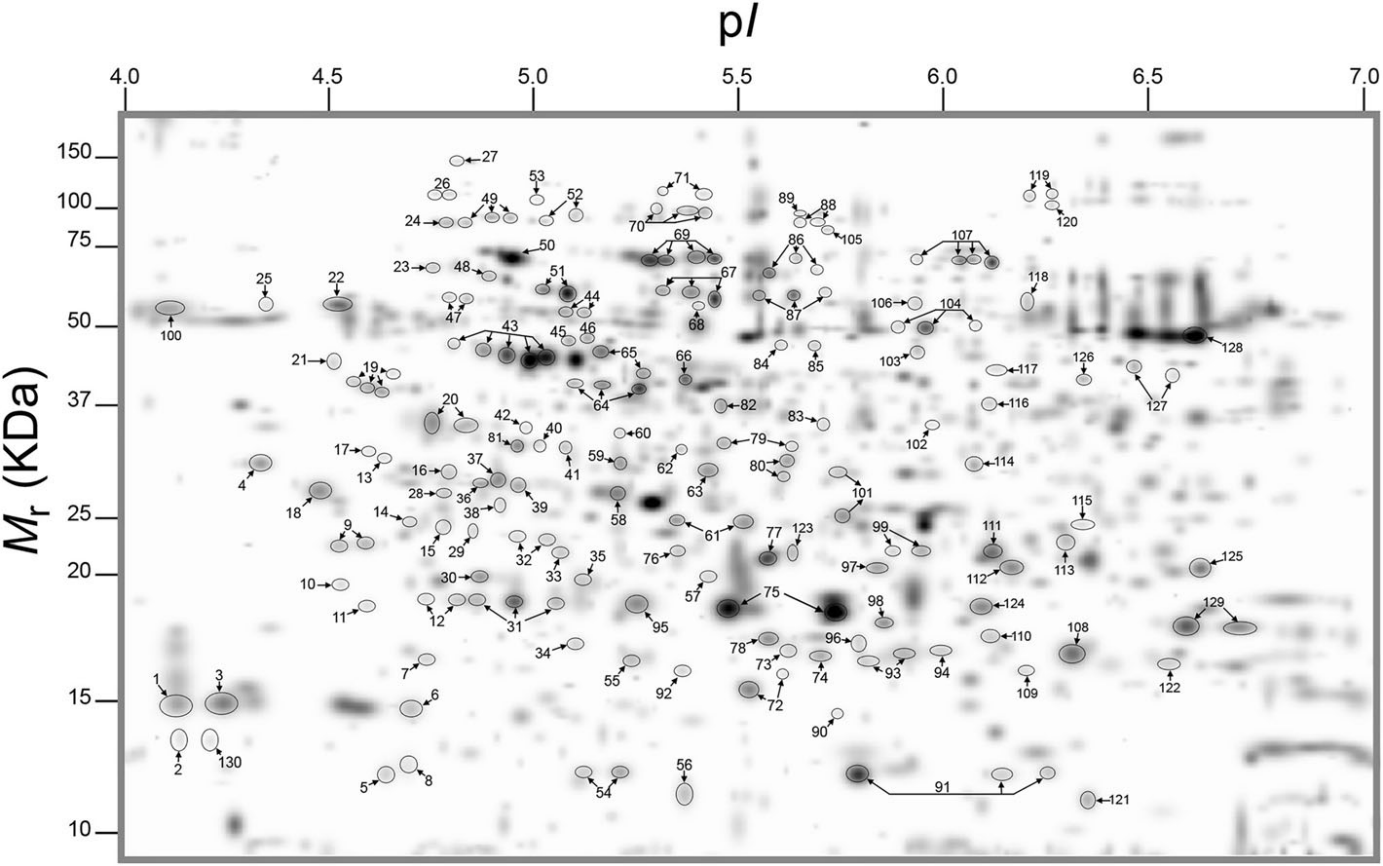
PDQuest-generated master gel image showing the general pattern of matched protein spots from the total leaf proteome of healthy or Las-infected grapefruit plants that were exposed or unexposed to thermal treatment of 40 °C for 6 days. Arrows point to protein spots that were differentially produced in response to Las-infection. Each differentially-expressed protein spot was assigned a unique number between 1 and 130. T*M*_r_, relative molecular weight; p*I*, isoelectric point

Nonetheless, a high resolution of total protein separation in a 4–7 p*I* range and 10–150 kDa molecular mass was observed in 2-DE gels of total leaf proteins from citrus grapefruit plants (Fig. 2). Mass spectrometry analysis identified 183 out of 188 protein spots that were differentially-expressed in citrus grapefruit leaves in response to Las infection and/or heat treatment. Multiple protein spots matched to the same protein, which could be due to a variety of factors including multimerism/protein isoforms, difference in maturation state, degradation and/or post-translational modifications [29, 30]. Thus, based on identical protein matches and proximity of spots on gels, the 183 identified spots were summarized into 130 protein spots (Fig. 3), and the MS-generated matched peptide sequences of the summarized 130 protein spots are provided in Additional file 1.

**Fig. 4:**
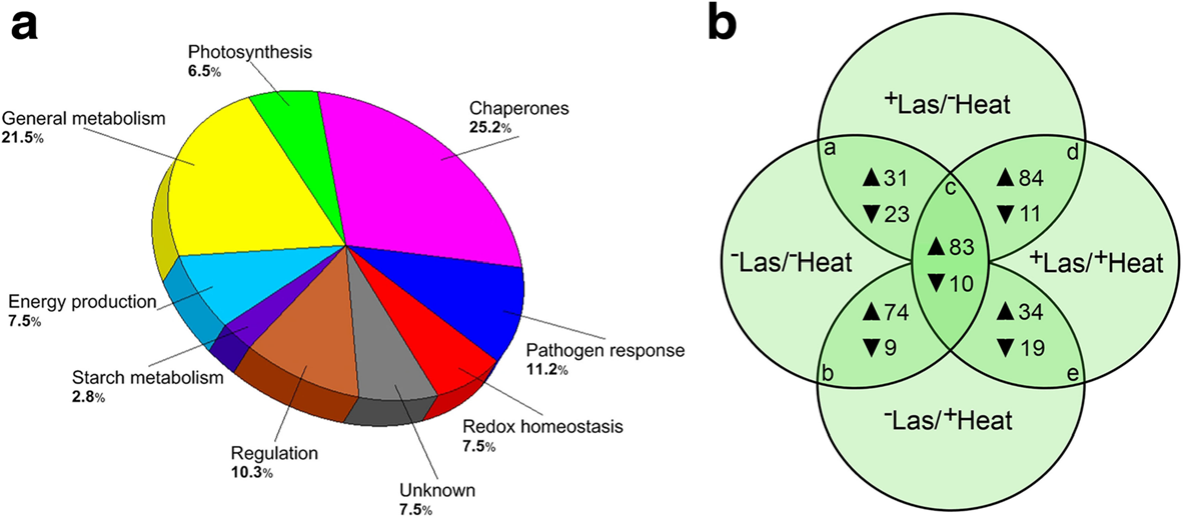
Categories of proteins that were up- or down-regulated in response to Las infection and/or heat treatment. **a** Functional category distribution of all identified differentially produced protein spots from comparing 2-DE gel images of the total leaf proteome of healthy or Las-infected grapefruit plants that were either unexposed or exposed to thermal treatment of 40 °C for 6 days. **b** Venn diagram with intersections a, b, c, d, and e, showing the number of identified protein spots that were significantly up- (▲) or down- (▼) regulated in (a) ^+^Las/^−^Heat plants compared to ^−^Las/^−^Heat plants; (b) ^−^Las/^+^Heat plants compared to ^−^Las/^−^Heat plants; (c) ^+^Las/^+^Heat plants compared to ^−^Las/^−^Heat plants; (d) ^+^Las/^+^Heat plants compared to ^+^Las/^−^Heat plants; (e) ^+^Las/^+^Heat plants compared to ^−^Las/^+^Heat plants

According to their expression patterns, sequence homology and functional similarities, the differentially-expressed protein spots were matched to 107 unique proteins and categorized into eight functional groups, namely: chaperones, pathogen response- and redox homeostasis-related proteins, in addition to proteins involved in photosynthesis, regulation, starch metabolism, energy production, and general metabolism (Fig. 4a). Chaperone-related proteins (for example heat shock proteins) constituted the largest functional group of proteins, accounting for over 25 % of all differentially-expressed proteins identified in this study (Fig. 4a). Additionally, about 7.5 % of the differentially-expressed proteins matched to uncharacterized proteins or proteins with yet to be determined functions (Fig. 4a).

Among the 107 differentially-expressed proteins, the volumes of 54 proteins significantly changed (31 up-regulated and 23 down-regulated) in ^+^Las/^−^Heat plants compared to ^−^Las/^−^Heat plants, showing the effect of Las treatment alone on protein expression. The effect of heat treatment alone on protein expression was highlighted by the 74 and 9 proteins that were up-and down-regulated, respectively, in ^−^Las/^+^Heat plants compared to ^−^Las/^−^Heat plants (Fig. 4b). The volumes of 93 proteins changed (83 up-regulated and 10 down-regulated) in ^+^Las/^+^Heat plants compared to ^−^Las/^−^Heat plants, denoting the combined effects of Las infection and heat treatment on citrus grapefruit plants (Fig. 4b). Further comparisons revealed an up-regulation of 84 proteins but down-regulation of 11 proteins in ^+^Las/^+^Heat plants compared to ^+^Las/^−^Heat plants, while 34 and 19 proteins were up- and down-regulated, respectively, in ^+^Las/^+^Heat plants compared to ^−^Las/^+^Heat plants (Fig. 4b).

### Chaperones displayed major heat-induced response to Las in citrus plants

**Fig. 5:**
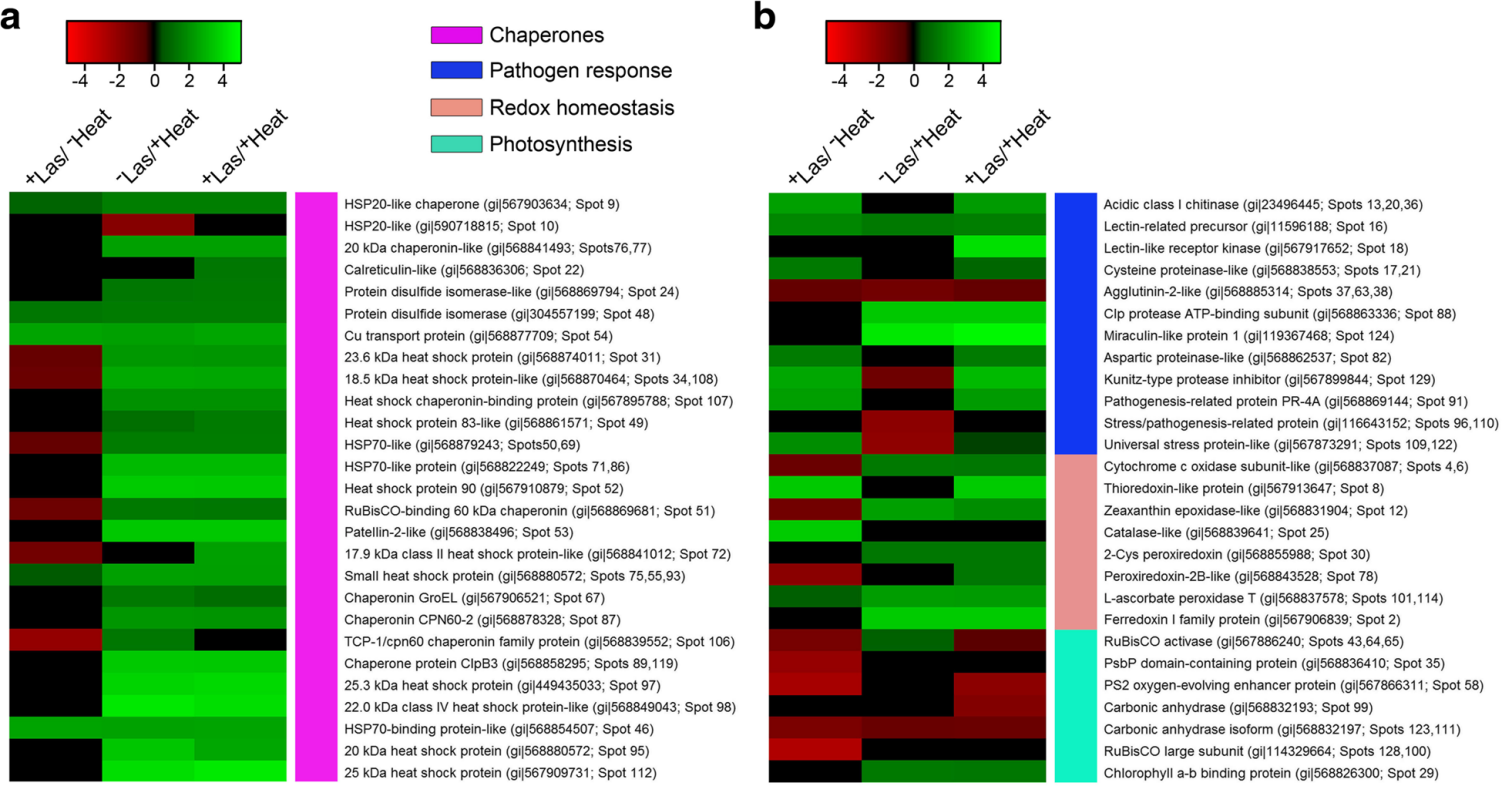
Differentially-expressed proteins from comparing 2-DE gel images of the total leaf proteome of healthy or Las-infected grapefruit plants unexposed or exposed to thermal treatment. **a** Chaperone-related proteins that were differentially-expressed in ^+^Las/^−^Heat, ^−^Las/^+^Heat or ^+^Las/^+^Heat plants compared to ^−^Las/^−^Heat plants. **b** Pathogen response-, redox homeostasis-, and photosynthesis-related proteins that were differentially-expressed in ^+^Las/^−^Heat, ^−^Las/^+^Heat or ^+^Las/^+^Heat plants compared to ^−^Las/^−^Heat plants. Red-black-green color schemes within columns represent relative fold changes normalized to a −5 to 5 range scale denoting the most down-regulated (bright red) to the most up-regulated (bright green) proteins. Black denotes no significant fold change for the given protein. The color-coded side bars correspond to functional groups of differentially-expressed proteins

Chaperones constituted the largest functional group of differentially-expressed proteins identified in this study (Fig. 4a). In ^+^Las/^−^Heat plants compared to ^−^Las/^−^Heat plants, 55 % or six out of the 11 differentially-expressed chaperone-related proteins, which included small (23.6, 18.5 and 17.9 kDa) heat shock proteins, a HSP70-like protein and a ribulose-1,5-bisphosphate carboxylase oxygenase (RuBisCO)-binding 60 kDa chaperonin, were down-regulated (Fig. 5a). However, in^−^Las/^+^Heat plants compared to ^−^Las/^−^Heat plants, 96 % or 24 out of the 25 differentially-expressed chaperone-related proteins were up-regulated (Fig. 5a). Subsequently, in ^+^Las/^+^Heat plants compared to ^+^Las/^−^Heat plants, there was an up-regulation of 20 chaperone-related proteins, which included a 20 kDa chaperonin-like protein, small (23.6, 18.5 and 17.9 kDa) heat shock proteins, a HSP70-like protein, HSP90 protein, chaperonin GroEL, and a RuBisCO-binding 60 kDa chaperonin (Fig. 5a). However, in ^+^Las/^+^Heat plants compared to ^+^Las/^−^Heat plants, there was no significant difference in the expression of four chaperone-related proteins including a HSP20-like chaperone and protein disulfide isomerase (Fig. 5a). This suggests that the 20 differentially-expressed chaperone-related proteins in ^+^Las/^+^Heat plants compared to ^+^Las/^−^Heat plants, could play a role in heat-mediated resistance to Las in citrus plants.

Molecular chaperones are stress response proteins involved in protein folding, refolding, assembly, re-assembly, degradation and translocation [31–34]. A prior study by our group showed that Las infection caused a broad down-regulation of chaperone-related proteins in grapefruit plants [28]. Citrus tristeza virus (CTV) exhibits a pathosystem similar to Las, and a proteomic study by Laino et al. [35] showed that CTV-tolerant citrus plants generally over-activate the phosphorylation of RuBisCO-binding proteins, chaperones and other reactive oxygen scavenging enzymes. It was, therefore, not surprising to observe that majority of the differentially-expressed chaperone-related proteins in ^+^Las/^−^Heat plants compared to ^−^Las/^−^Heat plants were down-regulated (Fig. 5a). On the other hand, chaperones are generally associated with stress response in plants and are typically up-regulated by heat stress [36], which is congruent with the observation of a general up-regulation of chaperone-related proteins due to heat treatment alone (Fig. 5a). Interestingly, compared to ^−^Las/^−^Heat plants, six chaperone-related proteins, including small (23.6, 18.5 and 17.9 kDa) heat shock proteins, a HSP70-like protein and a RuBisCO-binding 60 kDa chaperonin, that were down-regulated in ^+^Las/^−^Heat plants, became up-regulated in ^−^Las/^+^Heat plants and/or ^+^Las/^+^Heat plants. Thus, proteomics results suggest that these six chaperone-related proteins may play important roles in heat-induced response to Las in citrus plants.

### Pathogenesis-related proteins actively involved in heat-induced mitigation of HLB

In ^+^Las/^−^Heat plants compared to ^−^Las/^−^Heat plants, 87.5 % or seven out of the eight differentially-expressed pathogen response-related proteins, including an acidic class I chitinase, a lectin-related precursor, a pathogenesis-related PR-4A protein, and a kunitz-type protease inhibitor, were up-regulated (Fig. 5b). In contrast, heat treatment alone (i.e. in ^−^Las/^+^Heat plants compared to ^−^Las/^−^Heat plants) resulted in the down-regulation of 57 % or four out of seven differentially-expressed pathogen response-related proteins (Fig. 5b). However, in ^+^Las/^+^Heat plants compared to ^+^Las/^−^Heat plants, three proteins including a lectin-related precursor, a Clp protease ATP-binding subunit and a miraculin-like protein 1 were up-regulated (Fig. 5b). This suggests a potential active role in Las suppression for these three proteins because seven other pathogen-response related proteins, including an acidic class I chitinase, a cysteine proteinase-like protein, an aspartatic proteinase-like protein, a pathogenesis-related PR-4A protein and a universal stress-protein, which were differentially-expressed in ^+^Las/^−^Heat plants compared to ^−^Las/^−^Heat plants and in ^+^Las/^+^Heat plants compared to ^−^Las/^−^Heat plants, were not differentially expressed in ^+^Las/^+^Heat plants compared to ^+^Las/^−^Heat plants (Fig. 5b).

Canonical pathogen response-related proteins, which include pathogenesis-related (PR) proteins [37], chitinases [38], lectin-like proteins [39, 40], miraculin-like proteins [41], proteinases, and proteinase inhibitors [42–44], are defense-related proteins that are typically induced by plants against pathogen attack. Currently, PR proteins are grouped into 17 independent families, PR-1 to PR-17, and PR-4 family consists of class I and class II chitinases, which differ by the presence (class I) or absence (class II) of a conserved N-terminal cystein-rich domain corresponding to the hevein protein, a small antifungal protein first isolated from rubber tree (*Hevea brasiliensis*) latex [45]. Lectin-like proteins, which are structurally and evolutionarily-related to agglutinin-like proteins [46], are involved in vascular tissue differentiation [47], but also play a role in pathogen resistance by plugging phloem sieve plates to prevent systemic spread of pathogens [40]. Kim et al. [48] and Achor et al. [49] showed that the accumulation of a lectin-like protein, at the sieve plates is associated with blockage of the translocation stream in HLB-affected citrus plants. Additionally, studies have demonstrated that lectin-like proteins interact with RNA molecules and are involved in long-distance trafficking, suggesting a role for these proteins in long-distance signaling response in HLB-affected citrus plants [39, 50].

Miraculin is a plant protein that can modify a sour taste into a sweet taste and offsets the acidic taste in fruits [51, 52]. Although characteristically expressed in fruits, the induction of miraculin-like proteins in non-fruit tissues (i.e. stems, leaves or roots) has been strongly associated with pest or pathogen attack, suggesting their involvement in defense [42, 53, 54]. Tsukuda et al. [41] first characterized two distinct miraculin-like proteins in rough lemon (*Citrus jambhiri* Lush), RlemMLP1 (miraculin-like protein 1) and RlemMLP2 (miraculin-like protein 2) and demonstrated the induction of RlemMLP1 and/or RlemMLP2 by microbe attack.

### Redox homeostasis-related proteins involved in inducing the inhibitory effects of heat treatment on HLB

Redox homeostasis-related proteins are involved in the prevention of oxidative stress, which is induced by reactive oxygen species (ROS). ROS are by-products of electron transport and redox reactions from metabolic processes such as photosynthesis and respiration. More importantly, the production of ROS has been shown to be markedly increased under conditions of biotic or abiotic stress [55, 56].

In ^+^Las/^−^Heat plants compared to ^−^Las/^−^Heat plants, three out of six differentially-expressed redox homeostasis-related proteins were down-regulated, including a putative cytochrome C oxidase, zeaxanthin epoxidase-like protein, and a peroxiredoxin 2B-like protein (Fig. 5b). On the other hand, in ^+^Las/^+^Heat plants compared to ^+^Las/^−^Heat plants, all seven identified differentially-expressed redox homeostasis-related proteins were up-regulated including a putative cytochrome C oxidase, zeaxanthin epoxidase-like protein, and a peroxiredoxin 2B-like protein that were initially down-regulated in the presence of Las infection alone (Fig. 5b). Furthermore, a 2-cys peroxiredoxin and a ferredoxin I family protein, which were not differentially-expressed in ^+^Las/^−^Heat plants compared to ^−^Las/^−^Heat plants, were found to be up-regulated in presence of heat (i.e. ^+^Las/^+^Heat plants compared to ^+^Las/^−^Heat plants) or in ^+^Las/^+^Heat plants compared to ^−^Las/^−^Heat plants (Fig. 5b).

While the roles of redox homeostasis-related proteins like peroxidases, peroxiredoxin, cytochrome C oxidase, zeaxanthin epoxidase and catalase in HLB development in citrus plants are yet to be established, Monavarfeshani et al. [57] showed an increase in the expression of protein disulfide isomerase, glutathione reductase, and Cu/Zn superoxide dismutase (SOD) was higher in Mexican lime trees in response to *Candidatus* Phytoplasma aurantifolia. Additionally, Doria et al. [58] identified the upregulation of SOD, catalase and peroxidases in sweet orange plants infected with CTV. The differential expression of ascorbate peroxidase and peroxiredoxins have been previously associated with the response of citrus grapefruit plants to Las infection [28] as well as the response of *Citrus sinensis* plants to *Xanthomonas axonopodis* pv. citri and non-host pathogen *Xanthomonas oryzae* pv. Oryzae [59]. Silencing of Arabidopsis AtCOX17-1 gene decreased the expression of genes involved in the response of plants to different stress conditions, including several genes that are induced by mitochondrial dysfunctions [60]. Zeaxanthin epoxidase catalyzes the interconversion of carotenoids zeaxanthin to violaxanthin [61]. Gholampour et al. [62] showed an upregulation of zeaxanthin epoxidase gene transcripts in Las infected grapefruit plants in the late stages of HLB and proposed that the upregulation of zeaxanthin epoxidase a photosynthetic response to protect the grapefruit photosynthesis system against Las.

Taken together, these results suggest a role for redox homeostasis-related proteins and highlights the key active proteins in this class that a potentially involved in inducing the inhibitory effects of heat treatment on HLB.

### Photosynthesis/CO_2_ assimilation-related proteins altered by Las with or without heat treatment

In the presence of Las infection alone (i.e. in ^+^Las/^−^Heat plants compared to ^−^Las/^−^Heat plants), all five differentially-expressed photosynthesis-related proteins, which included RuBisCO activase, PS2 oxygen-evolving enhancer protein, and a RuBisCO large subunit protein, were down-regulated (Fig. 5b). However, in the presence of heat treatment alone (i.e. in ^−^Las/^+^Heat plants compared to ^−^Las/^−^Heat plants), two of the three differentially-expressed photosynthesis-related proteins, which included RuBisCO activase and chlorophyll a/b binding protein were up-regulated (Fig. 5b). Among the seven photosynthesis-related differentially-expressed proteins, while there was no difference in the expression of RuBisCO activase and PS2 oxygen-evolving enhancer protein in ^+^Las/^+^Heat plants compared to ^+^Las/^−^Heat plants, a PsbP domain-containing protein and a Chlorophyll a/b binding protein were found to be up-regulated in ^+^Las/^+^Heat plants compared to ^+^Las/^−^Heat plants (Fig. 5b). This suggests an active role for the PsbP domain-containing protein and Chlorophyll a/b binding protein in heat-mediated mitigation of HLB in citrus plants.

Chlorophyll a/b binding proteins form part of the light harvesting complex proteins [63] and a PsbP domain-containing protein was found to be essential for photosystem I assembly in *Arabidopsis* [64]. Although the role of PsbP domain-containing proteins or Chlorophyll a/b binding proteins in heat-mediated alleviation of HLB is yet to be resolved, transcripts of chlorophyll a/b binding protein were found to be up-regulated in *Citrus auratifolia* plants in response to CTV infection [65], suggesting a potential role for Chlorophyll a/b binding proteins in the mitigation of diseases caused by phloem-restricted pathogens.

### Regulatory-related proteins generally upregulated during heat-induced mitigation of HLB

**Fig. 6:**
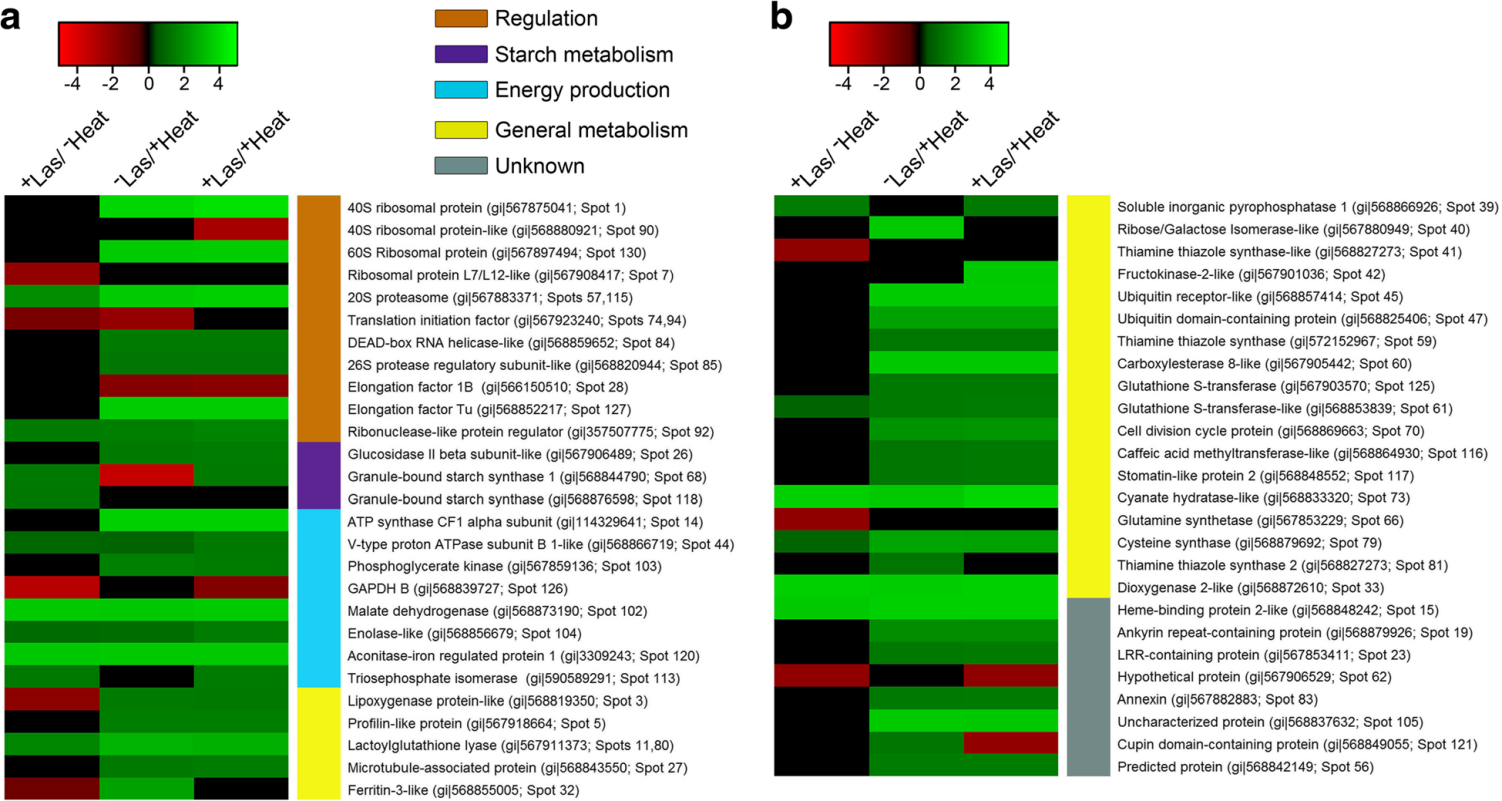
Differentially-expressed proteins from comparing 2-DE gel images of the total leaf proteome of healthy or Las-infected grapefruit plants unexposed or exposed to thermal treatment. **a** Regulation-, starch metabolism-, energy production-, and general metabolism-related proteins that were differentially-expressed in ^+^Las/^−^Heat, ^−^Las/^+^Heat or ^+^Las/^+^Heat plants compared to ^−^Las/^−^Heat plants. **b** Continuation of general metabolism-related proteins as well as functionally-uncharacterized proteins that were differentially-expressed in ^+^Las/^−^Heat, ^−^Las/^+^Heat or ^+^Las/^+^Heat plants compared to ^−^Las/^−^Heat plants. Red-black-green color schemes within columns represent relative fold changes normalized to a −5 to 5 range scale denoting the most down-regulated (bright red) to the most up-regulated (bright green) proteins. Black denotes no significant fold change for the given protein. The color-coded side bars correspond to functional groups of differentially-expressed proteins

Regulation-related proteins, for example a 20S proteasome and a ribonuclease-like protein regulator, were generally up-regulated in the presence of Las and/or heat treatment compared to ^−^Las/^−^Heat plants (Fig. 6a). Subsequently, a 40S ribosomal protein, 60S ribosomal protein, DEAD-box RNA helicase-like protein, 26S protease regulatory subunit-like protein, and elongation factor Tu, were up-regulated in ^+^Las/^+^Heat plants compared to ^+^Las/^−^Heat plants (Fig. 6a).

Proteasomes are multi-subunit and multi-catalytic agents that function as gene expression modulators responsible for most of the cytosolic and nuclear protein degradation via the ubiquitin-dependent or ubiquitin-independent proteolytic pathways. Laino et al. [35] identified a proteasome subunit α-type protein that was up-regulated in CTV-susceptible sour orange rootstocks grafted with Taracco plants upon CTV infection but did not observe a similar response in CTV-tolerant Carrizo citrange rootstocks grafted with Taracco plants upon CTV infection. Furthermore, a proteasome subunit α-type protein was upregulated in sweet orange mutants with higher antioxidant activity than wild-type plants [66]. Taken together, this suggests that proteasomes might play a role in the induction of plant-microbe incompatibility processes associated with the suppression of Las titer in citrus tissues under heat treatment.

### Starch metabolism

The accumulation of starch in plant tissues during Las infection has been well documented [67, 68]. Nwugo et al. [69] highlighted an inverse relationship between photosynthesis and starch anabolism processes whereby Las-mediated accumulation of starch suppresses the photosynthetic machinery via a negative feed-back effect. Thus, consistent with the results from previous studies, the present study showed an up-regulation of granule-bound starch synthase in Las-infected plants irrespective of heat exposure (Fig. 6a).

However, in the presence of heat treatment there was an up-regulation of a glucosidase II beta subunit-like protein (Fig. 6a), which is noteworthy because glucosidases are typically associated with starch catabolism but have been previously implicated in conferring disease resistance in plants. Cherif et al. [70] showed that an increase in beta-glucosidase activity in cucumber roots was associated with silicon-induced resistance to *Pythium* spp. Another study demonstrated that a soybean beta-glycosidase related to the *Lotus japonicus* defense gene, a-hydroxynitrile glucosidase, suppresses the parasitic activities of the nematode *Meloidogyne incognita* [71]. Furthermore, Miche et al. [72] identified a putative endo-1,3-beta-D-glucosidase as part of the jasmonate-induced defense-responsive proteins in the roots of rice plants upon exposure to the endophyte *Azoarcus* sp. Strain BH72.

Thus, the up-regulation of glucosidase in heat treated plants compared to non-heat treated plants might play a role in heat-mediated molecular mechanisms responsible for the reversal of Las pathogenesis processes in citrus plants, providing a potentially viable target for genetic engineering of HLB-resistance in citrus plants.

### Energy production-related proteins generally upregulated during heat-mediated HLB resistance

Protein production is an energy intensive process. Thus, considering the high number of unique proteins with up-regulated expression levels due to heat treatment (Fig. 4b), it was not surprising that under heat treatment, several energy production-related proteins, including a phosphoglycerate kinase protein, malate dehydrogenase and an aconitase-iron regulated protein were generally up-regulated (Fig. 6a). However, out of the eight energy production-related proteins that were differentially-expressed in response to Las and/or heat treatment, only two proteins, an ATP synthase CF1 alpha subunit and phosphoglycerate kinase were up-regulated in ^+^Las/^+^Heat plants compared to ^+^Las/^−^Heat plants (Fig. 6a).

While more experiential information is necessary to delineate the specific roles of energy production/TCA cycle-related proteins during heat-mediated HLB mitigation in citrus, in Las-infected Navel orange plants, isopropyl malate isomerase, which converts citrate to isocitrate and pyruvate decarboxylase involved in fermentation, were up-regulated in response to Las infection [73]. Additionally, the similarities between the Las pathosystem and the viral-based CTV pathosystem have been previously highlighted and plant RNA viruses have been shown to use host metabolic enzymes and housekeeping proteins in ways unrelated to their original functions [74]. Chloroplast phosphoglycerate kinase, a gluconeogenic enzyme, was shown to up-regulate *Bamboo mosaic virus* multiplication in *Nicotiana benthamiana* [75]. On the other hand, ATP synthase-γ subunit and Rubisco activase were respectively found to negatively regulate the movement and accumulation of the Tobacco mosaic virus in *Nicotiana tabacum* [76].

### General metabolism-related proteins differentially-expressed in response to Las infection and/or heat treatment

In the presence of Las infection alone (i.e. in ^+^Las/^−^Heat plants compared to ^−^Las/^−^Heat plants), four out of ten differentially-expressed general metabolism-related proteins, including a lipoxygenase-like protein and a ferritin-like protein, were down-regulated (Fig. 6a and b). However, in the presence of heat treatment alone (i.e. in ^−^Las /^+^Heat plants compared to ^−^Las/^−^Heat plants), all 19 differentially-expressed general metabolism-related proteins were up-regulated, including a putative lipoxygenase protein, a ferritin-like protein, and a glutathione S-transferase (Fig. 6a and b). Interestingly, ^+^Las/^−^Heat plants compared to ^−^Las/^−^Heat plants ^+^Las/^+^Heat plants, a ferretin-like protein, glutamine synthetase and a thiamine thiazole synthase-like protein were down-regulated but the same proteins were not down-regulated in ^+^Las/^+^Heat plants compared to ^−^Las/^−^Heat plants, suggesting a heat-mediated reversal of the expression of these proteins. Additionally, 12 proteins including lipoxygenase-like protein, thiamine thiazole synthase, glutathione S-transferase, a cell division cycle protein, and a stomatin-like protein were upregulated in ^+^Las/^+^Heat plants compared to ^+^Las/^−^Heat plants (Fig. 6b).

Ferritins are multimeric iron storage proteins suggested to be part of an iron-withholding defense system induced by hosts in response to bacterial invasion [77]. By using *Arabidopsis thaliana* as a susceptible host for the pathogenic bacterium *Erwinia chrysanthemi* Dellagi et al. [78] showed that ferritin accumulation during infection of *Arabidopsis* by *E. chrysanthemi* is a basal defense mechanism which is mainly activated by bacterial siderophores. A subsequent study by Liu et al. [79] reported that during pathogen attack, reactive Fe^3+^ accumulates in the cell walls of maize plants leading to intracellular iron depletion, which promotes the transcription of pathogenesis-related genes including ferritins. Thus, since Las infection resulted in the down-regulation of a ferritin-like protein, the lack of any difference in ferritin-like protein expression in ^+^Las/^+^Heat plants compared to ^−^Las/^−^Heat plants suggests a reversal of Las-mediated processes and highlights a potential target mechanism associated with heat-mediated mitigation of HLB.

Among the heat-induced general metabolism-related proteins identified, lipoxygenase and glutathione S-transferase are noteworthy since these proteins have been previously implicated in plant response to pathogens. Gardiner [80] found that the up-regulation of glutathione S-transferase contributes to the defense response of barley to trichothecenes, a major group of toxins produced by phytopathogenic fungi, including *Fusarium graminearum*. However, the disruption of a maize root-expressed 9-lipoxygenase gene was shown to enhance resistance to the anthracnose leaf blight pathogen *Colletotrichum graminicola* due to the constitutive activation of induced systemic resistance signaling. Hence, the roles of the heat-induced general metabolism-related proteins identified in the present study, including lipoxygenase and glutathione S-transferase, requires further substantiation.

## Conclusions

HLB, which is etiologically-linked to Las, is currently the most destructive disease of citrus and all commercially grown citrus species/relatives are susceptible to the disease. However, some citrus species, for example lemon plants, have demonstrated relatively high levels of tolerance to Las and it was recently shown that heat treatment or thermotherapy triggers host defensive response to Las infection. These observations suggest that citrus plants might possess innate HLB tolerance/resistance processes, which are inducible by abiotic factors, for example heat. The present study identified 107 proteins that were differentially expressed in response to Las and/or heat treatment, which included chaperones, pathogen response- and redox homeostasis-related proteins, in addition to proteins involved in photosynthesis, regulation, starch metabolism, energy production, and general metabolism. Among these proteins, chaperones including small (23.6, 18.5 and 17.9 kDa) heat shock proteins, a HSP70-like protein and a RuBisCO-binding 60 kDa chaperonin were strongly up-regulated by heat treatment. Additionally, chlorophyll a/b binding protein, glucosidase II beta subunit-like protein, a putative lipoxygenase protein, a ferritin-like protein, and a glutathione S-transferase were found to be down-regulated by Las infection but up-regulated in the presence of heat treatment, highlighting molecular mechanisms potentially involved in reversing the effects of Las infection in citrus plants. Thus, due to the absence of any known HLB resistance genes in cultivated *Citrus* spp., it is anticipated that the information generated from the present study would facilitate the development of cisgenic Las-resistant or tolerant citrus plants.

## Methods

### Growth conditions and treatments

Healthy and Las-positive citrus trees were prepared in the USHRL greenhouse, and used in this study as previously described [14]. HLB-affected trees were generated via side-grafting with three to four centimeter Las-positive bud sticks to clean Duncan grapefruits (*Citrus paradisi*) a year prior to the experiment. Both healthy and infected trees were maintained in the psyllid-proof greenhouse at the U.S. Horticulture Research laboratory, in Fort Pierce, Florida. Plants were irrigated as needed and fertilized every 3 weeks as previously described [14]. Plants were confirmed to be either healthy or Las-infected based on the disease symptoms present and Las titer as determined by real-time PCR [81]. Three-year-old similarly-sized healthy and Las-infected plants were either grown at room temperature (RT) or exposed to thermal treatment of 40 °C for 144 h (6 days) in a growth chamber (Conviron CMP5000, Winnipeg, Canada), with fluorescent lamps at 40 % intensity, a 12-h photoperiod, and 85 % relative humidity. Three replicate plants were used per treatment. A mixture of leaf tissues, both unhardened and hardened flush were collected from each plant at Time 0 h (before commencement of heat treatment) and Time 144 h (6 days after commencement of heat treatment). Harvested leaves were collected from plants exposed to heat treatment as well as plants not exposed to heat treatment and were immediately frozen in liquid nitrogen and stored at −80 °C until further analysis.

### Measurement of Las titer in plant tissues

Harvested leaves were ground to a fine powder in liquid nitrogen using a freezer mill (6850 Freezer/Mill, Wolf Laboratories Ltd., UK). DNA isolation and measurement of Las titer from heat treated and non-heat plant tissues were performed as previously described by Hoffman et al. [14]. Briefly, total genomic DNA was extracted from 0.1 g of ground leaf tissues using the CTAB method [82]. Extracted DNA from individual plants was quantified (Quant-iT™ PicoGreen^®^ dsDNA Assay Kit, Life Technologies, USA) and standardized to 30 ng μL^−1^. With the isolated genomic DNA used as template, forward (5′-CGGTGAATGTATTAAGCTGAGGCGTTCC-3′) and reverse (5′-TACCCACAACAAAATGAGATACACCAACAACTTC-3′) primers designed to amplify a segment of an “elongation factor Ts” sequence locus within the Las genome [83], were used for qPCR analysis to compare Las titer in leaf tissues across treatments. When necessary, the same forward and reverse primers for “elongation factor Ts” of Las was used for conventional PCR analysis to confirm the systemic presence or absence of Las in experimental plants. An endogenous citrus actin gene was used as the control/normalizer as previously described [84].

### Protein extraction and quantification

Harvested leaves from individual plants were ground to a fine powder in liquid nitrogen using a freezer mill (6850 Freezer/Mill, Wolf Laboratories Ltd., UK). Protein extraction methods have shown discrepancies in total protein coverage and each method tends to isolate a distinct “extractome” [85]. Hence, to enhance total protein coverage, an exhaustive protein extraction process involving two extraction methods, TCA acetone method after Nwugo et al. [69] and phenol extraction method after Wang et al. [86], were employed concurrently and the final products from both methods were combined prior to further analysis.

For TCA acetone extraction, 0.5 g of ground leaf tissues were suspended in 4.5 mL of chilled solution A [90 % (v/v) acetone, 9.9993 % (v/v) trichloroacetic acid (TCA), 0.0007 % (v/v) Beta-mercaptoethanol] and incubated overnight at −80 °C followed by centrifugation at 4 °C for 20 min at 36,000 *g* (Optima L-70 K Ultracentrifuge, Beckman Coulter Inc., USA). The pellet was washed three times by resuspension in 4.5 mL of chilled solution B [98.53 % (v/v) acetone, 1 mM polymethylsulphonylfluoride (PMSF), 2 mM EDTA, 0.0007 % (v/v) Beta-mercaptoethanol], incubation for 1 h at −80 °C followed by centrifugation at 4 °C for 20 min at 36,000 *g*. The pellet or crude protein extract was vacuum-dried (Vacufuge™, Eppendorf, Germany) and solubilized in 0.5 mL of rehydration/isoelectric focusing (IEF) buffer [8 M Urea, 50 mM DTT, 4 % (w/v) CHAPS, 0.2 % (v/v) 3/10 ampholytes, 0.002 % (w/v) bromophenol blue].

For phenol extraction, approximately 0.1 g of ground leaf tissues were suspended in 800 μL of SDS extraction buffer [30 % (w/v) sucrose, 2 % SDS, 0.2 mM EDTA, 0.1 M Tris HCl (pH 8), 5 % (v/v) Beta-mercaptoethanol and 2 mM PMSF] by vortexing continuously at 4 °C for 1 h. Additional 800 μL of phenol (saturated with Tris HCl, pH 8) was added and mixed briefly before centrifugation at 10, 000 *g* for 10 min at 4 °C. The top/phenol phase was retained and mixed with 5 volumes of 0.1 mM ammonium acetate in methanol, followed by incubated at −80 °C for 45 min and centrifugation at 18, 000 *g* for 20 min at 4 °C. The pellet was washed once in ice cold 100 % methanol and twice in ice cold 100 % acetone by resuspension and centrifugation at 18, 000 *g* for 20 min at 4 °C. The pellet or crude protein extract was air-dried and solubilized in 0.1 mL of IEF buffer.

For accurate quantification of extracted proteins from TCA acetone or phenol extraction methods, 5 μL of solubilized proteins were first treated with the Compat-Able™ Protein Assay Preparation Reagent kit (Pierce, Rockford, IL, USA) to remove interfering substances prior to bicinchoninic acid (BCA) assay (Pierce, Rockford, IL, USA). The total protein extraction process via TCA acetone and phenol as well as the quantification process was repeated three times generating three analytical replicates per plant. Total protein concentration was adjusted to 1.5 mg mL^−1^ for all samples. The corresponding analytical replicates of total leaf proteins isolated using TCA acetone or phenol extraction were combined 1:1 (v/v) prior to two-dimensional electrophoresis (2-DE) analysis.

### 2-DE and gel-image analysis

The separation of total extracted proteins was achieved by 2-DE as previously described [69]. Briefly, 300 μg of extracted soluble proteins from each sample was loaded on an 11-cm long pH 4–7 IpG strip and separated according charge via isoelectric focusing. Focused proteins in gel strips were further separated according to size on an SDS-PAGE gel and stained with Coomassie Brilliant Blue to generate 2-DE gel images. The PDQuest software (version 7.3.0, Bio-Rad, USA) was used to detect spots that had ≥10-fold increase over background and present in at least six of the nine gels per treatment. Detected spots that showed >1.5-fold change (*P* < 0.05) in volume/intensity across treatment groups were considered to be differentially produced and excised for mass spectrometry-based identification.

### Mass spectrometry and protein identification

Excised protein spots were trypsin-digested as previously described [69] and analyzed via liquid chromatography-assisted mass spectrometry (Eksigent nanoLC 1D-plus pump and Autosampler AS-2 attached to a NanoFlex cHiPLC™ that was interfaced to a QSTAR Elite QTOF-MS system by a NanoSpray® II electrospray ionizer, ABSCIEX, USA). Briefly, Tryptic-peptides dissolved in Reagent A (0.1 % formic acid in water, Sigma, USA) were loaded on to a cHiPLC™ silica trap-column (200 μm X 0.5 mm packed with ChromXP C18-CL of 3 μm bead size and 300 Å pore size, Eksigent Technologies, USA) and washed with Reagent A for 10 min. The retained peptides were separated on a cHiPLC™ silica analytical column (75 μm X 15 cm packed with ChromXP C18-CL of 3 μm bead size and 120 Å pore size, Eksigent Technologies, USA) using the following optimized sequence for Reagent B (0.1 % formic acid in acetonitrile, Sigma, USA): linear gradient from 12 to 35 % in 35 min, 95 % for 10 min, and 5 % for 5 min. To prevent sample carry-over, 100 % isopropanol was processed as “blank” in-between actual samples using the following sequence for Reagent B: 5 to 35 % in 3 min, 95 % for 17 min, and 5 % for 5 min. All LC analyses were performed at 40 °C column temperature at a flow rate of 0.3 μL min^−1^.

For each information dependent acquisition (IDA) cycle, one full TOF MS from 400 to 1600 m/z was scanned in positive mode with a resolution of 12,000 and an accumulation time of 1 s followed by Product Ion scans from 100 to 1600 m/z at low Quad resolution setting monitoring 3 most intense peaks with a maximum accumulation time of 3 s. A total duration time for data acquisition was set for 49 min with 10 s per cycle. An exclusion list of known contaminants was used to refine the MS spectrum and MS/MS target ions were excluded every 12 s. An AutoCal script (ABSCIEX, USA) was activated for automated internal calibration of mass spectra using the triple-charged (737.7067) and double-charged (1106.0562) monoisotopic peaks of a common trypsin auto-digestion product (MH^+^: 2211.42 Da) as calibrants.

For protein identification, the MASCOT search engine and Mascot Daemon (Matrix Science, London, UK) was used to automate searches of MS/MS fragmentation spectra first against a custom database containing entries for citrus (*Citrus sinensis* and *Citrus Clementina*) available at https://www.citrusgenomedb.org. This was accompanied by searches against entries for all plant species available in the NCBI nonreduntant database (https://www.citrusgenomedb.org). The PAC numbers for citrus genes or Accession numbers for other plant species that generated the highest Mascot score and percent peptide coverage against our protein/peptide queries were recorded. Fixed and variable modifications (Cys carbamidomethylation and Met oxidation, respectively) and one missed cleavage were considered. Peptides with charges of 2^+^ and 3^+^ were selected with a peptide mass tolerance of ± 50 ppm for MS scans, while a parent ion tolerance of 0.1 Da was selected for MS/MS scans. A decoy search was done automatically on a randomized database of equal composition and size to limit false detection rate. To gain functional information on identified proteins, homology searches using BLAST_P_ (https://blast.ncbi.nlm.nih.gov/Blast.cgi?PROGRAM=blastp&PAGE_TYPE=BlastSearch&LINK_LOC=blasthome) was employed. In situations where multiple spots match to the same protein the spot volumes were averaged within each treatment group to represent the protein expression volume for that treatment group. However, when multiple inconsistently differentially-expressed spots match to the same predicted/putative protein, such spots were treated as unique proteins.

### Statistical analysis

Ct values from qPCR analysis were subjected to analysis of variance (ANOVA) using SigmaPlot software Version 11 (Systat Software, Inc., California, USA) and means were separated using the Holm-Sidak method at ˃ 95 % confidence interval (*P* < 0.05). Proteomic analysis was performed on plant tissues harvested at time 144 h. Tissues were divided into four treatment groups as follows: ^−^Las/^−^Heat, representing healthy plants with no heat exposure; ^+^Las/^−^Heat, representing infected plants with no heat exposure; ^−^Las/^+^Heat, representing healthy plants with heat exposure; ^+^Las/^+^Heat, representing infected plants with heat exposure. Pair-wise comparisons to determine significant differences in spot intensities between treatments were performed on standardized log_10_ values of protein spot volumes using the Student’s *t*-test analysis at ˃ 95 % confidence interval (*P* < 0.05) as provided by the PDQuest™ 2-DE Analysis Software (Bio-Rad, Inc., California, USA). The R package was used to generate heat maps based on the fold change in protein production in leaves of ^−^Las/^−^Heat plants compared to ^+^Las/^−^Heat, ^−^Las/^+^Heat or ^+^Las/^+^Heat plants.

## Additional file

Additional file 1: Appendix 1. Mascot match results and peptide sequences of the 130 differentially-expressed spots presented in Fig. 3. (XLSX 3295 kb)

## Abbreviations

2-DE: Two dimensional electrophoresis
ANOVA: Analysis of variance
BCA: Bicinchoninic acid
BLAST: Basic local alignment search tool
CHAPS: 3-[(3-cholamidopropyl) dimethylammonio]-1-propanesulfonate
CTV: Citrus tristeza virus
DTT: Dithiothreitol
EDTA: Ethylenediaminetetraacetic acid
HLB: Huanglongbing
HSP70: Heat shock proteins70
IDA: Information dependent acquisition
IEF: Isoelectric focusing
kDa: Kilodalton
Las: ‘*Candidatus* Liberibacter asiaticus’
NCBI: National Center for Biotechnology Information
pI: Isoelectric point
PMSF: Polymethylsulphonylfluoride
PR: Pathogenesis-related
qRT-PCR: Quantitative real time polymerase chain reaction
ROS: Reactive oxygen species
RuBisCO: Ribulose-1,5-bisphosphate carboxylase
SOD: Superoxide dismutase
TCA: Trichloroacetic acid
TCA cycle: Tricarboxylic cycle
TOF MS: Time-of-flight mass spectrometry
